# Research participation effects: a skeleton in the methodological cupboard^☆^

**DOI:** 10.1016/j.jclinepi.2014.03.002

**Published:** 2014-08

**Authors:** Jim McCambridge, Kypros Kypri, Diana Elbourne

**Affiliations:** a Faculty of Public Health & Policy, London School of Hygiene & Tropical Medicine, 15-17 Tavistock Place, London WC1H 9SH, UK; b Centre for Clinical Epidemiology and Biostatistics, School of Medicine and Public Health, University of Newcastle, HMRI Building, Callaghan NSW 2308, Australia; c Department of Medical Statistics, Faculty of Epidemiology and Population Health, London School of Hygiene & Tropical Medicine, Keppel Street, London WC1E 7HT, UK

**Keywords:** Research participation, Bias, Research methods, Hawthorne effect, Research assessment, Mixed methods, Surveys, Cohort studies

## Abstract

**Objective:**

There have been concerns about impacts of various aspects of taking part in research studies for a century. The concerns have not, however, been sufficiently well conceptualized to form traditions of study capable of defining and elaborating the nature of these problems. In this article we present a new way of thinking about a set of issues attracting long-standing attention.

**Study Design and Setting:**

We briefly review existing concepts and empirical work on well-known biases in surveys and cohort studies and propose that they are connected.

**Results:**

We offer the construct of “research participation effects” (RPE) as a vehicle for advancing multi-disciplinary understanding of biases. Empirical studies are needed to identify conditions in which RPE may be sufficiently large to warrant modifications of study design, analytic methods, or interpretation. We consider the value of adopting a more participant-centred view of the research process as a way of thinking about these issues, which may also have benefits in relation to research methodology more broadly.

**Conclusion:**

Researchers may too readily overlook the extent to which research studies are unusual contexts, and that people may react in unexpected ways to what we invite them to do, introducing a range of biases.

**What is new?**

• “Research participation effects” offer a new way of thinking about poorly understood sources of bias in surveys and cohort studies, and also in trials.
• Research studies are unusual contexts, and people may react in unexpected ways to what we invite them to do.
• Adopting the perspective of the participant suggests that existing well-known sources of bias may be connected to each other.
• Mixed methods participant-centred research may lead to better prevention of bias.

The construct of “research participation effects” (RPE) has been proposed to better guide the empirical investigations of issues previously conceptualized as the Hawthorne effect [1]. We have also elaborated overlooked implications for behavioral intervention trials, identifying mechanisms by which bias may be introduced which randomization does not prevent [2]. This discussion considers the wider implications of RPE for thinking about bias, particularly addressing existing thinking about bias in surveys and cohort studies.

New ways of understanding biases provide platforms for important advances in research design and methods. For example, Solomon [3] identified that the discovery of “pre-test sensitisation”, whereby measuring individual psychology or behavior at one point of time biased later measurement of the same characteristics, led to the introduction of control groups within behavioral sciences. Chalmers [4] identified allocation concealment to prevent selection bias as the primary motivation for the use of randomization in the original streptomycin trial. Chalmers [4] has suggested that addressing biases resulting from patient preferences may provide the next historical milestone in the development of trials methodology. Just as patients may prefer allocation to one arm of a clinical trial over another, people may react to whatever it is they are requested to do in the context of research. These reactions have the potential to affect study outcomes in ways that undermine the validity of inferences the research was designed to permit.

A few years after the Hawthorne effect made its debut in the scientific literature [5], the concept of “demand characteristics” was introduced to psychology [6]. This referred to the ways in which study participants responded to their perceptions of the implicit preferences of researchers, tailoring their responses so as to be good subjects. Like the Hawthorne effect, although being well known, this construct has contributed disappointingly little to the methodological literature [7]. The unintended effects of research assessments have received attention other than when conceptualized as the Hawthorne effect. Randomized evaluation studies often show small effects, though there are inconsistencies [8], [9], [10], [11], [12].

Change due to having been assessed, having views about the desirability of different possible research requirements, and deliberately or unwittingly trying to satisfy researchers, are all consequences of research participation. The interaction of the research participant with the research process is discernible as a common thread running through these examples. The consequences of research participation may vary in strength across study designs, participants, topic areas, and the contexts in which research is done, and according to more specific features of the studies themselves.

## 1 Well-established biases in surveys and cohort studies

Ensuring adequate response rates, that is securing participation itself, is widely established as a key issue in survey design [13]. Evidence has accumulated over decades on how to do this [14], and in a context of falling response rates there has been extensive research on the implications of non-response for the estimation of prevalence and other parameters of interest in general household surveys [13]. There has also been much study of reporting errors made by participants in surveys, which draws attention to the sensitivity of the particular behavior or issue being enquired about [15]. This literature also distinguishes between task-related errors that are technical products of survey design, and motivated responses, for example, in the form of self-deception and impression management [16]. Thus in surveys, biases associated with research participation apply both to the decision to take part and to the accuracy of information provided. These biases may be conceptualized in many ways and often are thought about differently across disciplines and over time [17].

In a prospective cohort or longitudinal study [18], repeated data collection permits consequences of research participation to manifest themselves in altered behavior, cognitions, or emotions [12]. As Solomon [3] described, it is possible for inferences about data collected at one time point to be biased simply because of earlier data collection. This complication is more likely to occur, and is more likely to be problematic, in certain circumstances (see below). Some outcomes cannot be influenced by reactivity to evaluation, for example, where data collection is unobtrusive [19].

Asking someone how often they ride a bicycle may increase cycling in some circumstances and not others. It can only do so if the causal pathway to this outcome involves behavior that can be modified by this procedure [20]. For example, if a study participant owns a bicycle and is asked about their cycling behavior or views about cycling in a cohort study of health and lifestyle, they might think further about cycling, and might cycle more frequently as a result. This would artificially inflate levels of cycling in the cohort. If the study participant does not have access to a bicycle, this is less likely to occur unless they first acquire the means to start cycling. Asking about cycling in a different context may also reduce the likelihood of this occurring. The psychological processes involved are not important here; the point is that the more such effects occur, the more they may undermine the objectives of the study by introducing bias.

This problem may not emanate only from the content of data collection. Participants may have read the consent form carefully and thought about their health and lifestyle before deciding whether or not to take part. A cohort study is thus vulnerable to both the possible reporting and participation problems previously described for cross-sectional surveys, at both study entry and at follow-up. Additionally, actual change in the behavior being investigated may have been induced. Change in the object of the evaluation influenced by any aspect of research participation entails bias, regardless of how it has been produced. This is so unless an assumption is made that such influences do not vary in time with repeated measurements, which is unlikely to be very often a safe assumption. Randomized controlled trials are cohort studies with randomization, and as such are vulnerable both to the previously described problems, and also to additional ones associated with randomization [2]. This implies problems in making valid inferences from research data that afflict all study designs. These problems are mostly, but not all, very well known. What is novel about this presentation is the suggestion that they are linked, and by extension that conceptualizing them in this way as RPE may lead to better understanding of methodological problems.

## 2 A research participant-centred perspective

**Fig. 1:**
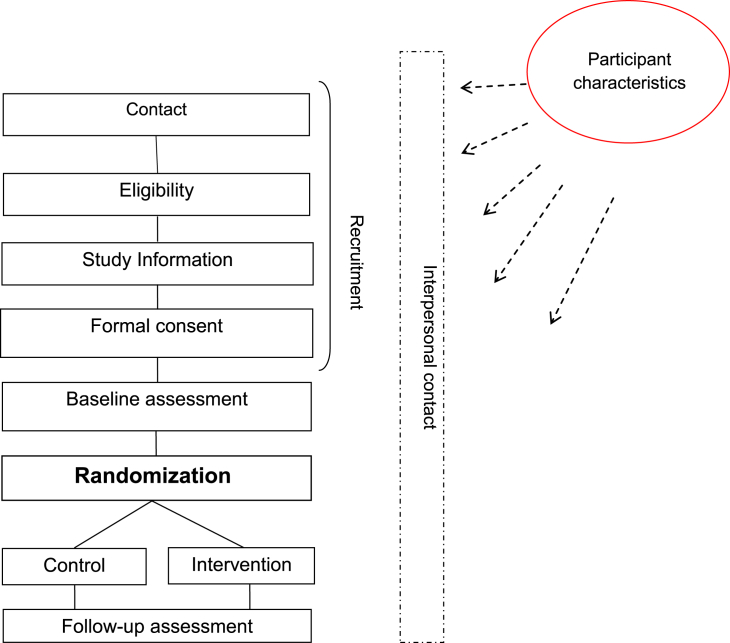
The research process. Cross-sectional surveys end with baseline assessment, cohort studies also involve follow-up assessment(s) only, RCTs involve randomization to study conditions as described previously. RCT, randomized controlled trial.

Different types of studies make different requests of, and place different demands on, their participants. There is nonetheless a core sequence of early events involving both a recruitment and baseline assessment phase, as presented in Fig. 1 for a typical individually randomized trial. We have found this a useful vehicle for thinking through the potential for RPE. For those who continue to participate over time, our lack of attention to the possible impact of the research process might imply that it is inert [12] and perhaps also that participants are somehow passive in this sequence. Fig. 1 provides a brief description of what we usually do to or with the people who become our research participants and in which order. It offers no information on participant characteristics or how or why they may matter to RPE. We suggest there is a *prima facie* case that reasons for participation, severity of problems or views about the issue being investigated, susceptibility to social desirability or monitoring effects, and readiness for change can all have a bearing on whether any of this process will impact on participants. These intrapersonal features might be expected to engage dynamically in the interpersonal process through which research participation is enacted. Research questions might address any of these targets for study.

Adopting a more participant-centred view of the research process [21] might first consider the nature of the decision-making involved in taking part in research [22]. Altruism has long been considered as the primary reason why people take part in most types of research [23]. Being disinterested in implications for self would appear to make RPE less likely, perhaps unless the research provides an unexpected stimulus for more personal introspection. More recent thinking has pointed toward more qualified versions of altruism, termed weak [24] or conditional [25] altruism, whereby a process of evaluation of the implications for oneself accompanies the motivation to help others in making decisions to take part in research. Such conditionality may be more likely in some circumstances than others. Trials and other intervention studies probably also attract those seeking interventions who are less altruistically minded for understandable reasons. Such a spectrum of reasons for participation may have implications for the generation of RPE, with less altruistic reasons more likely to generate RPE. There is little literature on participant reasons for continuation in research studies over time [26] and it may be profitable to pay attention to other influences on ongoing participation in cohort studies and trials [27].

Qualitative studies should be useful in identifying targets for study. There are studies available on many of the aspects of the research process already described, including for example how much prospective participants read and engage with provided study information [28], [29]. Study of preferences (see [30]) is another area where qualitative methods have uncovered problems within the largely quantitative endeavor that is randomized controlled trials. Preferences for allocation in trials have not only been found to exist, but also to be quite dynamic over time and capable of being influenced by dedicated interventions [31].

There are not studies, however, which evaluate individual participant-level qualitative data and also explore the possible implications for bias at the quantitative study level [27]. This is probably because there has not been an explicit effort to apply the type of conceptualization suggested here, which links qualitative and quantitative and individual and study level data. Beyond investigations of the acceptability of research procedures to prospective participants, there has been no programmatic approach to studying the effects of apparently mundane aspects of taking part in research. We offer an example that demonstrates that it is not difficult to do these types of studies and for participants to discuss their engagement with the research process; a qualitative study showing how thwarted preferences for allocation to a novel intervention led to disappointment and subsequently to movements both toward and away from change in a weight-loss trial [32].

This situation is perhaps not dissimilar to the 30-year tradition of study of participant cognitive engagement with surveys, where much quantitative and qualitative data have been used to enhance the content of particular surveys, but have yielded disappointing progress in methodology for questionnaire design [33]. Our perspective suggests that unrecognized potential for bias resides in routine research practice. We acknowledge that this calls for a type of mixed methods orientation [34] in which the core concepts and issues are framed as done in quantitative research, and that a qualitative phenomenological approach is used to identify possible problems, which may in turn be further evaluated in quantitative studies. What might be described as a post-positivist concern for bias adopted here may be unsatisfactory to some qualitative researchers who have epistemological differences with such an approach [35]. This may also be unfamiliar territory for many readers of an epidemiology journal, which we suggest is useful to explore for new insights into the nature of biases. In Box 1 we offer some suggestions for helpful questions to ask in a given study, and for developing this type of research more widely.

Box 1 **Helpful questions for researchers**

1. Why are participants taking part in this study?
2. What does taking part in this study mean for participants?
3. Why do participants behave as they do in this study?
4. How does what participants do affect any concerns about bias?
5. How far are the most likely sources of bias connected in this study?
6. Is existing thinking about bias adequate for the methodological problems faced here?
7. How might existing thinking about bias be extended to address methodological problems not well covered?
8. What can qualitative data or quantitative data contribute to better understand these issues?
9. How can qualitative data and quantitative data be combined to address research participation effects?
10. How can the construct of research participation effects be developed to guide more advanced study?

## 3 Conclusion

The potential for RPE may be intrinsic to all human research designs, though there are probably many areas where it can be safely ignored, as unlikely to threaten valid inference. There are other domains of research where they certainly cannot be ignored. The problem is that we do not know where this is the case, and therefore further conceptual work and empirical studies elaborating these issues are needed. We suggest that conventionally understood forms of bias as found in cross-sectional surveys and cohort studies are also interpretable as RPE. Furthermore, this preliminary conceptualization may be fruitful for creative thinking about biases and how to minimize them in designing research studies.

RPE is unwittingly created in the decisions made by researchers. Paying attention to the practices of researchers and approaching research on the research enterprise more sociologically [36] will also be useful. Because of their origins in the decisions made by researchers, RPE may be amenable to control in design, or in analysis if it is not possible to prevent them. Although we have known something of RPE for around 100 years [3], it will be disappointing if future progress is as slow as in the past. Perhaps this is partly because they call attention to unresolved and difficult-to-resolve issues to do with the relationship between quantitative and qualitative research approaches and data. RPE is nonetheless a skeleton in the methodological cupboard that deserves a decent burial.
